# El papel del IMC, las moléculas del perfil lipídico sérico y sus índices derivados en los pólipos colorrectales

**DOI:** 10.1515/almed-2024-0060

**Published:** 2024-05-20

**Authors:** Chunyu Huang, Weipeng Liang, Yuying Sun

**Affiliations:** State Key Laboratory of Oncology in South China, Guangdong Provincial Clinical Research Center for Cancer, 71067 Sun Yat-sen University Cancer Center, Guangzhou, China; Servicio de Endoscopias, 71067 Sun Yat-sen University Cancer Center, Guangzhou, China; Cancer Prevention Center, 71067 Sun Yat-sen University Cancer Center, Guangzhou, China

**Keywords:** índice de masa corporal, pólipo colorrectal, lípidos séricos

## Abstract

**Objetivos:**

Investigar el papel del IMC, las moléculas del perfil lipídico en suero y los cocientes lipoproteicos en los pólipos colorrectales.

**Métodos:**

En un análisis retrospectivo, se incluyó a 352 sujetos sometidos a una colonoscopia en nuestro centro, de los cuales 247 no mostraron ninguna alteración evidente (grupo control), mientras que 105 recibieron un diagnóstico de uno o múltiples pólipos (grupo de pacientes). Se compararon las moléculas del perfil lipídico sérico y los cocientes lipoproteicos de los dos grupos.

**Resultados:**

El grupo de pacientes mostró niveles significativamente mayores de colesterol total (CT) y apolipoproteína B (ApoB) que el grupo de control (p<0,05). Entre los hombres, el grupo de pacientes mostró niveles de ApoB y una relación ApoB/ApoA1 superiores a los del grupo de control (p<0,05). Así mismo, los niveles de triglicéridos (TG) y la relación TG/C-HDL (colesterol de lipoproteínas de alta densidad) fueron significativamente más elevados en el grupo de pólipos múltiples que en el de un solo pólipo (p<0,05). Además, los niveles de C-HDL y la relación C-HDL/ApoA1 fueron más altos en el grupo con pólipos adenomatosos que en el de no adenomatosos (p<0,05). El análisis de regresión logística múltiple identificó al CT, TG, LDL-C y a los cocientes CT/C-HDL, TG/C-HDL y C-LDL/C-HDL como factores de riesgo para el desarrollo de pólipos colorrectales (p<0,05). Los análisis de la curva ROC revelaron una asociación entre el CT, la ApoB, y la relación ApoB/ApoA1 y los pólipos colorrectales. Por otro lado, no se observaron diferencias estadísticamente significativas en el IMC entre los dos grupos (p>0,05).

**Conclusiones:**

La incidencia y evolución de los pólipos colorrectales están relacionados con las moléculas del perfil lipídico en suero y los cocientes lipoproteicos de las mismas. La dislipidemia podría incrementar el riesgo de desarrollar pólipos colorrectales, pudiendo derivar posteriormente en el desarrollo de cáncer colorrectal (CRC).

## Introducción

Los pólipos colorrectales se definen como el crecimiento excesivo de hiperplasias de la mucosa del colon y el recto. Las tasas de detección de pólipos colorrectales han aumentado significativamente, debido al incremento de las pruebas de cribado mediante colonoscopia y los cambios en el estilo de vida de la población. El adenoma colorrectal se considera una lesión precancerosa, ya que la secuencia conocida del cáncer colorrectal es “adenoma-hiperplasia adenomatosa atípica-carcinoma” [1, 2]. Los estudios demuestran que se pueden reducir los índices de mortalidad del cáncer colorrectal mediante la extracción temprana de los pólipos colorrectales [3]. De este modo, la identificación de factores de riesgo asociados a los pólipos colorrectales cobra especial relevancia a la hora de prevenir el cáncer colorrectal (CRC). No obstante, existe cierto debate sobre la relación entre los pólipos colorrectales y los niveles de lípidos séricos. Existe evidencia de que el síndrome metabólico está estrechamente relacionado con el desarrollo de pólipos colorrectales. Así mismo, la dislipidemia ha sido identificada como uno de los factores patogénicos del CRC [4], [5], [6], aunque otros estudios descartan la relación entre los lípidos séricos y los pólipos colorrectales [7, 8]. Los objetivos del presente estudio eran analizar retrospectivamente los niveles de las moléculas del perfil lipídico en suero y los cocientes lipoproteicos en pacientes con pólipos colorrectales y controles. Entre otros objetivos, se incluyó evaluar la correlación entre los niveles lipídicos individuales y los pólipos colorrectales, explorar los factores de riesgo de desarrollar pólipos colorrectales y establecer un marco teórico de referencia para el cribado de grupos con alto riesgo de desarrollar pólipos colorrectales. Los resultados del estudio también podrían ayudar a reducir la incidencia del CRC y mejorar la calidad de vida de los pacientes. Este estudio reveló que existe una relación entre los niveles lipídicos en suero y los pólipos colorrectales. La dislipidemia, definida como niveles elevados de colesterol total (CT), triglicéridos (TG), colesterol de lipoproteínas de baja densidad (C-LDL) y apolipoproteína B (ApoB), podría aumentar la probabilidad de desarrollar pólipos colorrectales. Del mismo modo, al analizar la relación entre los derivados lipídicos y los pólipos colorrectales, observamos una relación entre los cocientes CT/colesterol de lipoproteínas de alta densidad (C-HDL), TG/C-HDL, C-LDL/C-HDL y ApoB/Apolipoproteína A1 (ApoA1) y la presencia de los pólipos colorrectales. Los cocientes lipoproteicos de los componentes del perfil lipídico en suero podrían haber cambiado, aunque fueran normales, por lo que los cocientes lipídicos séricos son mejores predictores de patología que las moléculas del perfil lipídico en suero. Los resultados de las moléculas de perfil lipídico y sus cocientes se pueden obtener por inspección simple, que es un método cómodo, rápido, económico y fácil de repetir, con gran relevancia en la prevención del CCR.

## Materiales y métodos

### Población de pacientes

Entre enero de 2019 y diciembre de 2020, un total de 435 pacientes se sometieron a una colonoscopia y a seis análisis de lípidos en sangre en el Centro de Prevención del Cáncer del Sun Yat-sen University Cancer Center. La mayoría de estos pacientes acudieron voluntariamente a nuestro centro para realizarse una colonoscopia y creían no presentar ninguna patología previamente a dicha prueba, sino que, para ellos, únicamente se trataba de un chequeo médico ordinario. Sin embargo, se excluyó a 88 pacientes por presentar inflamación o divertículos en el colon, melenas, hiperplasia folicular linfática, lesiones submucosas o cáncer de colon precoz, entre otras patologías, por lo que la muestra total del estudio fue de 352 pacientes. De estos 352 pacientes, la presencia de pólipos se descartó mediante colonoscopia en 247 pacientes, que se incluyeron en el grupo de control. Los restantes 105 pacientes recibieron un diagnóstico de pólipos colorrectales y fueron asignados al grupo de pacientes. Así mismo, se crearon varios subgrupos de pacientes, en función de los resultados de la colonoscopia con respecto al diámetro, cantidad, localización y tipo histológico de los pólipos colorrectales. Se realizó en ambos grupos un análisis exhaustivo de las moléculas del perfil lipídico en suero y de los cocientes lipoproteicos. El grupo de pacientes se dividió en varios subgrupos, con el objeto de evaluar las moléculas del perfil lipídico en sangre, según las características clínicas de los pólipos colorrectales. Todos los participantes del estudio firmaron un formulario de consentimiento informado. El presente estudio fue aprobado por el Comité de Ética del Sun Yat-sen University Cancer Center y se ajusta a los principios de la Declaración de Helsinki.

Se realizó un análisis retrosepctivo de los datos clínicos de los pacientes, incluyendo el sexo, la edad, el peso, la altura, las características de los pólipos colorrectales (diámetro, cantidad, localización, tipo histológico de los pólipos colorrectales), las moléculas de perfil lipídico en suero y los cocientes lipoproteicos. Entre las moléculas individuales del perfil lipídico se incluyeron TG, CT, C-HDL, C-LDL, ApoA1 y ApoB, mientras que entre los cocientes lipoproteicos se incluyeron C-LDL/C-HDL, CT/C-HDL, TG/C-HDL, C-HDL/ApoA1 y el cociente ApoB/ApoA1. Entre las características de los pólipos analizadas se encuentran el diámetro, cantidad, localización y tipo histológico de los pólipos colorrectales.

A continuación, se detallan los criterios de exclusión: (1) antecedentes quirúrgicos de pólipos colorrectales y cáncer colorrectal; (2) otras enfermedades colorrectales, aparte de los pólipos colorrectales, como colitis, enfermedad colorrectal inflamatoria, enfermedad del colon, divertículos en el colon y cáncer colorrectal; (3) enfermedades primarias relacionadas con el metabolismo lipídico con ingesta de fármacos reguladores lípidicos y otros fármacos que puedan afectar a las moléculas del perfil lipídico sérico en los últimos 3 meses; (4) enfermedades cardíacas y cerebrales graves, así como disfunción hepática y renal.

### Colonoscopia

Las colonosocopias fueron realizadas por endoscopistas experimentados con un dispositivo electrónico OLYMPUS CF-HQ290. A todos los componentes del grupo de pacientes se les realizó una biopsia o se extrajo el pólipo para su posterior análisis anatomopatológico. Dos patólogos expertos examinaron las secciones histológicas.

Según el diámetro del o los pólipos, se asignó al grupo de pacientes al subgrupo <1 cm o al subgrupo ≥1 cm, mientras que, según el número de pólipos, se asignó a los pacientes al subgrupo de pólipo solitario o de múltiples pólipos. También se establecieron subgrupos según la localización del o los pólipos en el colon, a saber: hemicolon izquierdo formado por el ángulo esplénico, colon descendente y colon sigmoide, y hemicolon derecho por el ciego, colon ascendente, ángulo hepático y colon transverso. Según los tipos histopatológicos, se dividió a los pacientes en pólipos adenomatosos (adenoma tubular, adenoma velloso, adenoma tubular-velloso y adenoma serrado) y pólipos no adenomatosos (pólipos inflamatorios, pólipos hiperplásicos).

### Análisis de lípidos séricos

Se extrajeron muestras de sangre (3 mL) a través de la vena cubital de todos los participantes tras ayuno de entre 8 y 12 horas. Una vez separado el plasma, se analizaron los niveles de CT, TG, C-HDL, C-LDL, ApoA1 y ApoB mediante un autoanalizador bioquímico (Cobas 8000 c702, Roche Diagnostics GmbH, Mannheim, Alemania). Los intervalos de referencia de las moléculas del perfil lipídico sérico son: CT: 3,1–5,69 mmol/L; TG: 0,2–1,7 mmol/L; C-HDL: 1,16–1,42 mmol/L; C-LDL: 2,2–3,1 mmol/L; ApoA1: 1,2–1,6 g/L; y ApoB: 0,6–1,0 g/L. En función de los resultados de las moléculas de perfil lipídico sérico, se calcularon los cocientes lipoproteicos, entre los que se incluyen C-LDL/C-HDL, CT/C-HDL, TG/C-HDL, C-HDL/ApoA1 y ApoB/ApoA1.

### Análisis estadístico

El análisis de los datos se realizó con el programa estadístico SPSS 25.0 (SPSS Inc. Chicago, IL, EE.UU.). Previamente a realizar la prueba t de Student, se realizó un estudio de normalidad para comprobar la distribución de los datos. Los datos cuantitativos se expresan en medias±desviación estándar $\left(\bar{x}\pm s\right)$ .

La comparación entre los diferentes grupos se analizó mediante la prueba *t* de muestras independientes, asumiendo la normalidad en la distribución de los datos. Los factores de riesgo independientes asociados a la presencia de pólipos colorrectales se analizaron mediante el método de análisis de regresión logística múltiple y el análisis de la curva ROC. Un valor p <0,05 se consideró estadísticamente significativo.

## Resultados

### Análisis comparativo de las moléculas del perfil lipídico en suero

No se observaron diferencias en términos de edad o sexo entre el grupo de pacientes y el grupo de control. En el grupo de pacientes, había 55 hombres frente a 50 mujeres, con una edad media de 51,14±9,01. En el grupo de control, había 111 hombres y 136 mujeres, con una edad media de 49,74±7,10. Los niveles de CT y ApoB en el grupo de pacientes fueron significativamente superiores que en el grupo de control (p<0,05). Se observaron diferencias estadísticamente significativas en la relación ApoB/ApoA1 entre los dos grupos (p=0,002) (Tabla 1). En el grupo de pacientes, la ApoB (p=0,025) y la relación ApoB/ApoA1 (p=0,024) fueron significativamente superiores que en el grupo de control entre los varones. Sin embargo, no se hallaron diferencias estadísticamente significativas entre las mujeres (p>0,05) (Tabla 3). No se estableció ninguna relación entre el índice de masa corporal (IMC) y los pólipos colorrectales (p>0,05) (Tabla 1).

**Tabla 1:** Analisis univariante del grupo de pacientes y controles.

Parámetros	Grupo de pacientes (n=105)	Grupo control (n=247)	Valor T	Valor p
Sexo [masculino, %]	55 (52,4)	111 (44,9)	1,279	0,202
Edad ( $\bar{x}\pm s$ , año)	51,14±9,01	49,74±7,10	1,563	0,119
IMC ( $\bar{x}\pm s$ , kg/m^2^)	23,98±2,62	23,39±2,73	1,875	0,062
CT ( $\bar{x}\pm s$ , mmol/L)	5,75±0,98	5,51±0,97	2,159	0,032^a^
TG ( $\bar{x}\pm s$ , mmol/L)	1,60±0,98	1,53±2,35	0,314	0,754
C-HDL ( $\bar{x}\pm s$ , mmol/L)	1,46±0,41	1,49±0,44	−0,508	0,612
C-LDL( $\bar{x}\pm s$ , mmol/L)	3,49±0,84	3,44±0,84	0,581	0,562
ApoA1 ( $\bar{x}\pm s$ , mmol/L)	1,54±0,25	1,58±0,27	−1,216	0,225
ApoB ( $\bar{x}\pm s$ , mmol/L)	1,10±0,24	1,02±0,21	3,144	0,002^a^
CT/C-HDL ( $\bar{x}\pm s$ )	4,20±1,23	3,94±1,20	1,800	0,073
TG/C-HDL ( $\bar{x}\pm s$ )	1,30±1,14	1,23±2,58	0,285	0,776
C-LDL/C-HDL ( $\bar{x}\pm s$ )	2,56±0,87	2,46±0,81	1,032	0,303
C-HDL/ApoA1 ( $\bar{x}\pm s$ )	0,94±0,13	0,93±0,14	0,304	0,761
Apo-B/ApoA1 ( $\bar{x}\pm s$ )	0,74±0,21	0,67±0,19	3,111	0,002^a^

ApoA1, apolipoproteína A1; ApoB, apolipoproteína B; IMC, índice de masa corporal; C-HDL, colesterol de lipoproteínas de alta densidad; C-LDL, colesterol de lipoproteínas de baja densidad; CT, colesterol total; TG, triglicéridos. ^a^p<0,05.

### Relación entre las diferentes características clínicas de los pólipos colorrectales y las moléculas del perfil lipídico sérico

Hubo 105 casos en el grupo de pacientes, incluyendo 30 casos de ≥45 años, y 75 casos de <45 años; 58 casos con pólipos únicos, y 47 casos de pólipos múltiples; 18 casos con pólipos ≥1 cm, y 87 casos con pólipos <1 cm; 48 casos con pólipos localizados en el colon derecho, y 57 localizados en el colon izquierdo o recto; 64 casos con pólipos adenomatosos, y 41 pólipos no adenomatosos (Tabla 2). En los varones del grupo de pacientes, los niveles de ApoB y la relación ApoB/ApoA1 fueron significativamente superiores a los del grupo de control (p<0,05) (Tabla Suplementaria 1), mientras que no hubo diferencias significativas en las mujeres (p>0,05) (Tabla Suplementaria 2). En el subgrupo de pólipos múltiples, los niveles de TG y la relación TG/C-HDL fueron significativamente superiores a los del subgrupo de pólipos únicos (p<0,05) (Tabla 3). Los niveles de C-HDL y la relación C-HDL/ApoA1 fueron superiores en los pólipos adenomatosos a los de los pólipos no adenomatosos (p<0,05) (Tabla 4). Sin embargo, no se estableció ninguna relación entre los niveles lipídicos en el grupo de pacientes y la edad, el tamaño o la localización del pólipo (p>0,05). En términos generales, la localización y el tamaño de los pólipos no se asoció significativamente con la dislipidemia.

**Tabla 2:** Caracterísicas de los sujetos.

Características	n (%)
Grupo de pacientes	105 (100,0)

Sexo	
Hombres	55 (52,4)
Mujeres	50 (47,6)
Edad, años	
≤45	30 (28,6)
＞45	75 (71,4)
IMC	
Normal	52 (49,5)
Sobrepeso	47 (44,8)
Obesidad	6 (5,7)
Localización de los pólipos	
Hemicolon izdo/recto	57 (54,3)
Hemicolon dcho	48 (45,7)
Tamaño de los pólipos, mm	
≥10	18 (17,1)
<10	87 (82,9)
Patología de los pólipos	
Adenoma	64 (61,0)
No adenoma	41 (39,0)
Nº de pólipos	
Único	58 (55,2)
Múltiple	47 (44,8)

Grupo control	247 (100,0)

Sexo	
Hombres	111 (44,9)
Mujeres	136 (55,1)
Edad, años	
≤45	47 (19,0)
>45	200 (81,0)
IMC	
Normal	153 (61,9)
Sobrepeso	80 (32,4)
Obesidad	14 (5,7)

**Tabla 3:** Análisis univariante del subgrupo por número de pólipos en el Grupo de pacientes.

Parámetros	Grupo pólipos múltiples (n=47)	Grupo pólipo único (n=58)	Valor T	Valor p
CT, mmol/L	5,67±0,95	5,82±0,99	−0,759	0,450
TG, mmol/L	1,88±1,24	1,38±0,62	2,670	0,009^a^
C-HDL, mmol/L	1,39±0,43	1,52±0,38	−1,654	0,101
C-LDL, mmol/L	3,41±0,84	3,56±0,84	−0,914	0,363
ApoA1, g/L	1,50±0,27	1,57±0,23	−1,450	0,150
ApoB, g/L	1,11±0,26	1,10±0,23	0,061	0,951
Relación CT/C-HDL	4,45±1,48	3,99±0,96	1,894	0,061
Relación TG/C-HDL	1,65±1,47	1,03±0,68	2,851	0,005^a^
Relación C-LDL/C-HDL	2,69±0,98	2,46±0,76	1,320	0,190
Relación C-HDL/ApoA1	0,91±0,14	0,96±0,13	−1,808	0,074
Relación ApoB/ApoA1	0,77±0,24	0,72±0,19	1,190	0,237

ApoA1, apolipoproteína A1; ApoB, apolipoproteína B; C-HDL, colesterol de lipoproteínas de alta densidad; C-LDL, colesterol de lipoproteínas de baja densidad; CT, colesterol total; TG, triglicéridos. ^a^p<0,05.

**Tabla 4:** Análisis univariante del subgrupo Tipo Patológico en el Grupo de pacientes.

Parámetros	Pólipos adenomatosos (n=64)	Pólipos no adenomatosos (n=41)	Valor T	Valor p
CT, mmol/L	5,75±0,96	5,76±1,01	−0,098	0,922
TG, mmol/L	1,51±1,00	1,75±0,92	−1,241	0,217
C-HDL, mmol/L	1,53±0,42	1,36±0,36	2,086	0,039
C-LDL, mmol/L	3,49±0,80	3,51±0,91	−0,130	0,897
ApoA1, g/L	1,56±0,25	1,50±0,24	1,271	0,207
ApoB, g/L	1,09±0,24	1,13±0,26	−0,752	0,454
Relación CT/C-HDL	4,01±1,19	4,49±1,26	−1,979	0,051
Relación TG/C-HDL	1,18±1,16	1,49±1,10	−1,357	0,178
Relación C-LDL/C-HDL	2,45±0,81	2,74±0,94	−1,706	0,091
Relación C-HDL/ApoA1	0,96±0,14	0,90±0,11	2,617	0,010^a^
Relación ApoB/ApoA1	0,72±0,21	0,77±0,22	−1,276	0,205

ApoA1, apolipoproteína A1; ApoB, apolipoproteína B; C-HDL, colesterol de lipoproteínas de alta densidad; C-LDL, colesterol de lipoproteínas de baja densidad; CT, colesterol total; TG, triglicéridos. ^a^p<0,05.

### Análisis de factores de riesgo independientes para desarrollar pólipos colorrectales

El análisis de regresión logística múltiple de las moléculas del perfil lipídico sérico y los cocientes lipoproteicos, donde se utilizó la aparición de pólipos colorrectales como variable dependiente, reveló que el CT, los TG, y los cocientes C-LDL, CT/C-HDL, TG/C-HDL y C-LDL/C-HDL eran factores de riesgo para la aparición de pólipos colorrectales (p<0,05) (Tabla 5). El CT, los TG y la relación ApoB/ApoA1 también se relacionaron con los pólipos colorrectales, según los análisis de curvas ROC (Tabla 6). Se realizó un análisis de la curva ROC para todas las variables, incluidos el CT, TG, C-LDL, C-HDL, ApoA1, ApoB, y las relaciones CT/C-HDL, TG/C-HDL y ApoB/ApoA1. Nuestros hallazgos muestran que el CT, los TG, y la relación ApoB/ApoA1 mostró mayores niveles de sensibilidad y especifidad para estos resultados clínicos.

**Tabla 5:** Regresión logística múltiple de las moléculas lipídicas séricas en el Grupo de pacientes.

Parámetros	B	Error estándar	Wald	Valor p	OR	95 % IC
Edad ( $\bar{x}\pm s$ , año)	0,033	0,017	3,620	0,057	1,033	0,999–1,068
IMC ( $\bar{x}\pm s$ , kg/m^2^)	0,078	0,055	1,992	0,158	1,081	0,970–1,205
CT, mmol/L	8,454	2,092	16,333	<0,001^a^	4693,743	77,790–2,832×10^5^
TG, mmol/L	−3,459	1,310	6,976	0,008^a^	0,031	0,002–0,410
C-HDL, mmol/L	−6,325	4,811	1,729	0,189	0,002	0,000–22,280
C-LDL, mmol/L	−10,286	2,479	17,219	<0,001^a^	<0,001	0,000–0,004
ApoA1, g/L	−2,479	6,258	0,157	0,692	0,084	0,000–1,778×10^4^
ApoB, g/L	8,320	4,633	3,225	0,073	4105,273	0,468–3,605×10^7^
Relación CT/C-HDL	−8,168	2,603	9,845	0,002^a^	<0,001	0,000–0,047
Relación TG/C-HDL	3,153	1,370	5,299	0,021^a^	23,405	1,598–342,882
Relación C-LDL/C-HDL	8,108	3,045	7,092	0,008^a^	3321,874	8,508–1,297×10^6^
Relación C-HDL/ApoA1	0,375	11,000	0,001	0,973	1,455	0,000–3,355×10^8^
Relación ApoB/ApoA1	−0,268	6,481	0,002	0,967	0,765	0,000–2,513×10^5^

ApoA1, apolipoproteína A1; ApoB, apolipoproteína B; B, coeficiente de regresión; IMC, índice de masa corporal; C-HDL, colesterol de lipoproteínas de alta densidad; C-LDL, colesterol de lipoproteínas de baja densidad; OR, Odds ratio; CT, colesterol total; TG, triglicéridos; 95 % IC, intervalo de confianza al 95 %. ^a^p<0,05.

**Tabla 6:** Resultados del análisis de la curva ROC del valor diagnóstico de los índices lipoproteicos en los pólipos colorrectales.

Parámetros	AUC	SE	Valor p	IC 95 %	Punto de corte	Sensibilidad	Especificidad	Índice de Youden
CT	0,575	0,033	0,025^a^	0,510–0,641	61,250	0,371	0,765	0,137
TG	0,561	0,034	0,069	0,494–0,628	18,750	0,295	0,830	0,125
C-HDL	0,487	0,034	0,692	0,420–0,554	12,650	0,686	0,352	0,038
C-LDL	0,508	0,034	0,812	0,442–0,574	26,700	0,886	0,170	0,056
ApoA1	0,454	0,034	0,170	0,388–0,520	12,050	0,962	0,069	0,031
ApoB	0,602	0,034	0,002^a^	0,535–0,669	12,050	0,381	0,826	0,207
CT/C-HDL	0,562	0,034	0,065	0,495–0,630	52,150	0,229	0,899	0,127
TG/C-HDL	0,548	0,034	0,152	0,481–0,615	15,750	0,276	0,834	0,110
ApoB/ApoA1	0,598	0,034	0,003^a^	0,531–0,665	07,650	0,495	0,717	0,212

ApoA1, apolipoproteínaA1; ApoB, apolipoproteínaB; C-HDL, colesterol de lipoproteínas de alta densidad; C-LDL, colesterol de lipoproteínas de baja densidad; CT, olesterol total; TG, triglicéridos; IC 95 %, intervalo de confianza al 95 %; AUC, área bajo la curva; SE, standard error. ^a^p<0.05.

## Discusión

El CRC es uno de los tumores malignos más comunes, con índices de morbimortalidad persistentemente altos [9]. El CRC presenta un bajo índice de detección en el cribado de la población general, no existiendo un método sencillo y efectivo para el censo de la población. La prueba de detección de hemoglobina fecal es una prueba común no invasiva cada vez más utilizada en el mundo en los programas de cribado del cáncer colorrectal. Sin embargo, esta prueba no se realizazó en este estudio debido al escaso cumplimiento de los participantes. Aunque la endoscopia es la prueba de referencia para el diagnóstico del CCR [10], en China existe una grave escasez de endoscopistas experimentados, sumado al hecho de que la endoscopia es una prueba invasiva y costosa, a menudo rechazada por las personas asintomáticas. De este modo, resulta difícil realizar el cribado del CCR mediante endoscopia, y el único método potencialmente eficaz es el cribado de la población con alto riesgo de CCR. El objetivo del presente estudio es ayudar a identificar a la población con riesgo elevado de CCR mediante la identificación de factores de alto riesgo de pólipos colorrectales, y servir de guía para el cribado del CCR. Por lo tanto, el estudio de los factores de riesgo de pólipos colorrectales es de gran importancia para la reducción de la incidencia del CCR.

Existe evidencia de que la dislipidemia desempeña un papel crucial en la aparición y desarrollo de multitud de tumores, lo que la convierte en una posible diana de las terapias oncológicas [11]. Las principales características del tejido tumoral son que el cuerpo pierde el control, lo que deriva en una proliferación celular excesiva y, eventualmente, en la formación del cáncer. El crecimiento celular rápido requiere la adición de más colesterol, ya que el colesterol es el principal componente de la membrana celular [12, 13]. La dislipidemia y el cáncer colorrectal están estrechamente relacionados. Se ha observado que la dislipidemia es considerablemente más frecuente en las personas con CCR. Según una extensiva investigación retrospectiva de casos y controles realizada en China, la diabetes, la hiperlipidemia y la enfermedad inflamatoria intestinal están relacionadas de forma independiente con el riesgo de CCR múltiple [4]. Debido a la popularidad de la tecnología endoscópica, los índices de detección de los pólipos colorrectales han aumentado significativamente en los últimos años. Aunque los pólipos colorrectales están asociados a la edad, el sexo, el tabaquismo, el consumo de alcohol, la dieta rica en grasas, la obesidad, la diabetes, el síndrome metabólico y otros factores, la etiología y patogenia de los pólipos colorrectales siguen sin estar claras. Así mismo, según algunos estudios, existe una relación estrecha entre la dislipidemia y el adenoma colorrectal [14], [15], [16], [17], [18], [19], [20], [21], aunque aún no existen pruebas concluyentes sobre dicha relación. Mientras que algunos investigadores no consideran que exista ninguna relación entre la dislipidemia y el adenoma colorrectal [7, 8], otros lo consideran un factor de riesgo para la incidencia y progresión del mismo. Por otro lado, los pólipos y los lípidos séricos, han sido objeto de escasos estudios [22], [23], [24], [25]. Hallamos niveles significativamente mayores de CT y ApoB en los pacientes con pólipos colorrectales que en los que no los presentaban (Tabla 1), lo que demuestra que la dislipidemia está asociada al pólipo colorrectal, coincidiendo con los resultados de otros estudios previos.

Actualmente, no existe evidencia científica concluyente sobre la relación entre la dislipidemia y los pólipos colorrectales. En los estudios, se ha observado que los TG no solo pueden activar el factor de crecimiento similar a la insulina-1 y regular la actividad de la proteína K-ras, sino que también estimulan la proliferación anormal de las células epiteliales intestinales y promueven la transformación del adenoma colorrectal a un tumor [17]. Otros estudios, también sugieren que la dislipidemia activa la vía MAPK aumentando la actividad de las especies de oxígeno reactivo de las células del adenoma de colon, lo que deriva en una proliferación celular excesiva y favorece la progresión de los pólipos a tumores [26]. Niveles elevados de TG pueden provocar un aumento de los factores proinflamatorios, a la vez que reducen las citocinas inhibitorias inflamatorias, lo que afecta al crecimiento, apoptosis y proliferación de tumores y células estromales, promoviendo la carcinogénesis de los adenomas [27]. La dislipidemia también puede provocar estrés oxidativo y actuar paralelamente a la inflamación para provocar efectos carcinogénicos. Considerado un importante factor patogénico de multitud de enfermedades, el estrés oxidativo provoca la producción de más especies reactivas del oxígeno, peroxidación lipídica y la transformación de las macromoléculas lipídicas de la membrana celular en peróxido lipídico, causando daño tisular. Esto demuestra que el estrés oxidativo y la peroxidación lipídica están relacionadas, y que el grado de peróxido lipídico está asociado al colesterol sérico y los TG [28, 29]. Así mismo, el aumento de las moléculas del perfil lipídico en suero también puede modificar la excreción y circulación de ácido biliar, activar el mecanismo de absorción de ácido biliar o de inhibición del ácido butírico, aumentar el contenido de ácido biliar secundario en el intestino, dañar el ADN, provocar proliferación celular, crear un microambiente angiogénico, y favorecer la aparición de pólipos colorrectales [20].

Algunos estudios sugieren que los cocientes lipoproteicos están relacionados con la aparición y el desarrollo de algunos tipos de cáncer. Así, existe evidencia de que la relación ApoB/ApoA1 está asociada a un incremento de la incidencia global del cáncer en los hombres [30]. En dicho estudio, también se halló una asociación con la ApoB. Además, en lo que respecta a los pacientes con CCR, observaron una relación con la ApoB, pero no con la ApoA1, lo que coincide con nuestros hallazgos. Según Kimak et al. y Ma et al., la relación ApoB/ApoA1 ere más elevada en los pacientes con cáncer gástrico (CG) [31, 32]. El aumento de la relación C-LDL/C-HDL en suero previo a la cirugía es un factor de riesgo independiente del estadio de los ganglios tumorales y metástasis del CCR, estando estrechamente relacionado con el pronóstico del CCR [33]. Sin embargo, actualmente existen pocos estudios sobre la correlación entre los cocientes lipoproteicos y la aparición y el desarrollo de pólipos colorrectales. Por este motivo, analizamos la relación entre los pólipos colorrectales, los lípidos séricos y los cocientes lipoproteicos en función de su localización, tamaño, número y tipos histológicos. Los resultados del presente estudio revelan que la dislipidemia, definida según los niveles de TG, CT, C-LDL, ApoB y las relaciones CT/C-HDL, TG/C-HDL, C-LDL/C-HDL y ApoB/ApoA1, desempeña un papel importante en la aparición y el desarrollo de pólipos colorrectales. Este hallazgo concuerda con el estudio llevado a cabo por Isakov et al., que demostró la asociación entre una relación TG/HDL elevada y la presencia de pólipos serrados [34]. Además, analizamos la relación entre los pólipos colorrectales y los cocientes lipoproteicos séricos por subgrupos, no habiendo observado ninguna correlación significativa entre la dislipidemia y la edad, la localización o el tamaño de los pólipos. Según los análisis de regresión múltiple, el CT, los TG, el C-LDL y la ApoB podrían estar relacionados con un mayor riesgo de desarrollar pólipos colorrectales (p<0,05). Los análisis de las curvas ROC también revelaron una asociación entre el CT, la ApoB, y la relación ApoB/ApoA1 con los pólipos colorrectales. Dichos resultados coinciden con estudios anteriores [23].

Por otro lado, la relación entre los lípidos y el IMC en los pólipos colorrectales también fue objeto de nuestro estudio [34–36]. Los resultados obtenidos en estudios previos sobre la asociación entre el IMC y los lípidos no arrojaron resultados concluyentes. En el presente estudio, observamos que no existe una correlación significativa entre el IMC y los pólipos colorrectales.

Este estudio presenta algunas limitaciones. En primer lugar, se trata de un estudio unicéntrico, habiéndose empleado un tamaño de muestra relativamente pequeño. Dado que los sujetos de este estudio fueron seleccionados entre los pacientes que se sometieron a una colonoscopia para un chequeo rutinario, podría haberse producido un sesgo de selección. Además, la investigación actual sobre los factores de riesgo relacionados con los pólipos colorrectales aún se puede perfeccionar, por lo que sería necesario realizar estudios en muestras más amplias, con el fin de obtener resultados más exactos. Por todo ello, se puede considerar que estos resultados son preliminares.

En resumen, el presente estudio sugiere que la dislipidemia podría tener un papel importante en la aparición y el desarrollo de pólipos colorrectales, siendo un factor de riesgo independiente. Observamos que las moléculas del perfil lipídico en suero y los cocientes lipoproteicos se podrían emplear como factores de predicción de la presencia de pólipos colorrectales. Se deberían realizar colonoscopias a las personas con dislipidemia con mayor frecuencia que en la población general.

## Supplementary Material

Supplementary Material

## Material Suplementario

Este artículo incluye material suplementario (https://doi.org/10.1515/almed-2024-0060).
