# Supplementary material for: El papel del IMC, las moléculas del perfil lipídico sérico y sus índices derivados en los pólipos colorrectales
Source: Adv Lab Med. 2024 May 20;5(3):283–90. [Article in Spanish] doi: 10.1515/almed-2024-0060 (PMC11381628; doi:10.1515/almed-2024-0060)
Supplement: Supplementary file 1 — Supplementary Material [file j_almed-2024-0060_suppl_001.docx]

**Tabla suplementaria 1.** Análisis univariante de los hombres del grupo de pacientes frente al grupo de control.

| Parámetros | Grupo de pacientes (n=55) | Grupo de control (n=111) | Valor T | Valor p |
| --- | --- | --- | --- | --- |
| Edad ($\bar{x}\pm s,$año) | 49,49$\pm$9,13 | 48,59$\pm$7,77 | 0,660 | 0,510 |
| IMC ($\bar{x}\pm s,$kg/m^2^) | 24,47$\pm$2,71 | 24,24$\pm$2,65 | 0,542 | 0,589 |
| CT (mmol/L) | 5,70$\pm$0,77 | 5,49$\pm$0,99 | 1,429 | 0,155 |
| TG (mmol/L) | 1,87$\pm$1,15 | 1,70$\pm$3,24 | 0,367 | 0,714 |
| C-HDL (mmol/L) | 1,30$\pm$0,31 | 1,37$\pm$0,46 | -0,970 | 0,334 |
| C-LDL (mmol/L) | 3,50$\pm$0,66 | 3,44$\pm$0,86 | 0,444 | 0,658 |
| ApoA1 (g/L) | 1,45$\pm$0,22 | 1,49$\pm$0,28 | -1,039 | 0,300 |
| ApoB (g/L) | 1,14$\pm$0,20 | 1,06$\pm$0,21 | 2,265 | 0,025* |
| Relación CT/C-HDL | 4,62$\pm$1,20 | 4,28$\pm$1,26 | 1,666 | 0,098 |
| Relación TG/C-HD | 1,65$\pm$1,37 | 1,46$\pm$3,52 | 0,378 | 0,706 |
| Relación C-LDL/C-HDL | 2,83$\pm$0,79 | 2,69$\pm$0,87 | 1,045 | 0,298 |
| Relación C-HDL/ApoA1 | 0,89$\pm$0,11 | 0,90$\pm$0,12 | -0,664 | 0,508 |
| Relación ApoB/ApoA1 | 0,81$\pm$0,20 | 0,73$\pm$0,19 | 2,272 | 0,024* |

ApoA1= Apolipoproteína A1; ApoB= Apolipoproteína B; IMC= Índice de masa corporal; C-HDL= colesterol de lipoproteínas de alta densidad; C-LDL= colesterol de lipoproteínas de baja densidad; CT= colesterol total; TG= triglicéridos.

*p<0,05

**Tabla suplementaria 2.** Análisis univariante de mujeres del grupo de pacientes frente al grupo de control.

| Parámetros | Grupo de pacientes (n=50) | Grupo de control (n=136) | Valor T | *Valor p* |
| --- | --- | --- | --- | --- |
| Edad ($\bar{x}\pm s,$año) | 52,96$\pm$8,61 | 50,67$\pm$6,40 | 1,964 | 0,051 |
| IMC ($\bar{x}\pm s,$kg/m^2^) | 23,45$\pm$2,43 | 22,71$\pm$2,61 | 1,738 | 0,084 |
| CT (mmol/L) | 5,81$\pm$1,17 | 5,53$\pm$0,96 | 1,664 | 0,098 |
| TG (mmol/L) | 1,31$\pm$0,62 | 1,39$\pm$1,20 | -0,422 | 0,674 |
| C-HDL (mmol/L) | 1,64$\pm$0,43 | 1,59$\pm$0,40 | 0,801 | 0,424 |
| C-LDL (mmol/L) | 3,49$\pm$1,00 | 3,43$\pm$0,83 | 0,366 | 0,715 |
| ApoA1 (g/L) | 1,64$\pm$0,25 | 1,64$\pm$0,25 | -0,165 | 0,869 |
| ApoB (g/L) | 1,07$\pm$0,28 | 0,99$\pm$0,21 | 1,975 | 0,050 |
| Relación CT/C-HDL | 3,73$\pm$1,11 | 3,67$\pm$1,08 | 0,354 | 0,724 |
| Relación TG/C-HD | 0,92$\pm$0,66 | 1,04$\pm$1,41 | -0,553 | 0,581 |
| Relación C-LDL/C-HDL ratio | 2,26$\pm$0,87 | 2,28$\pm$0,72 | -0,110 | 0,912 |
| Relación C-HDL/ApoA1 | 0,99$\pm$0,13 | 0,96$\pm$0,14 | 1,428 | 0,155 |
| Relación ApoB/ApoA1 | 0,67$\pm$0,21 | 0,62$\pm$0,16 | 1,711 | 0,089 |

ApoA1= ApolipoproteínaA1; ApoB= ApolipoproteínaB; IMC= Índice de masa corporal; C-HDL= colesterol de lipoproteínas de alta densidad; C-LDL= colesterol de lipoproteínas de baja densidad; CT= colesterol total; TG= triglicéridos.

*p<0,05
